# An interpretable machine learning model integrating early immune biomarkers for predicting outcomes after spinal cord injury

**DOI:** 10.3389/fneur.2026.1855360

**Published:** 2026-09-08

**Authors:** Xuheng Jiang, Dandan Zhou, Ke Ma, Ji Zhang, Han Hu, Zhirui Xue, Mo Li, Anping Liu, Tianjing Sun, Lili Shi, Xiaofei Huang, Haizhen Duan, Tianxi Zhang, Xiuquan Shi, Hu Shengli, Anyong Yu

**Affiliations:** 1 Department of Emergency, Affiliated Hospital of Zunyi Medical University, Zunyi, Guizhou, China; 2 Department of Infectious Diseases, Affiliated Hospital of Zunyi Medical University, Zunyi, Guizhou, China; 3 Department of Epidemiology and Health Statistics, School of Public Health, Zunyi Medical University, Zunyi, Guizhou, China; 4 Center for Pediatric Trauma Research & Center for Injury Research and Policy, The Abigail Wexner Research Institute at Nationwide Children's Hospital, The Ohio State University College of Medicine, Columbus, OH, United States; 5 Department of Neurosurgery and Key Laboratory of Neurotrauma, Southwest Hospital, Third Military Medical University (Army Medical University), Chongqing, China

**Keywords:** immune-inflammation, neutrophils, prognosis, risk prediction model, spinal cord injuries

## Abstract

**Objective:**

To characterize early systemic immune-inflammatory alterations after acute spinal cord injury (SCI), evaluate their association with 12-month neurological outcomes, and develop an interpretable model for early risk stratification.

**Methods:**

This retrospective cohort study included 664 patients with acute traumatic injury. Patients were stratified according to vertebral fracture and SCI status to compare early peripheral immune-inflammatory profiles. Among them, 315 patients with SCI and complete 12-month follow-up data were included in the prognostic analyses. The primary outcome was an unfavorable neurological outcome, defined as death or an American Spinal Injury Association Impairment Scale (AIS) grade A–C at 12 months. Candidate predictors were identified using univariable logistic regression and least absolute shrinkage and selection operator (LASSO) regression, followed by multivariable logistic regression and Extreme Gradient Boosting (XGBoost) modeling. Model performance was evaluated using receiver operating characteristic (ROC) analysis, bootstrap validation, calibration analysis, decision curve analysis, and SHapley Additive exPlanations (SHAP).

**Results:**

Patients with SCI exhibited more pronounced early systemic immune-inflammatory activation, characterized by elevated white blood cell count (WBC), absolute neutrophil count (ANC), and neutrophil-related inflammatory indices, accompanied by reduced lymphocyte parameters and higher incidences of pneumonia and deep vein thrombosis. Among the 315 patients with SCI, 110 (34.9%) experienced unfavorable outcomes. These patients had greater injury severity, worse baseline neurological status, more frequent cervical cord involvement, higher inflammatory marker levels, and more complications. The final model incorporated Injury Severity Score (ISS), AIS grade A, cervical cord involvement, and ANC. Compared with AIS grade A alone, the multivariable model demonstrated improved discrimination (AUC, 0.958 vs. 0.886; *p* < 0.001), with good calibration and minimal optimism on bootstrap validation. The XGBoost model showed comparable performance (AUC, 0.943). SHAP analysis further supported the relative importance of the selected predictors.

**Conclusion:**

Acute SCI is associated with marked early neutrophil-dominant systemic inflammation, and elevated ANC was consistently associated with unfavorable neurological status at 12 months. A parsimonious model integrating ISS, AIS grade A, cervical cord involvement, and ANC provided additional prognostic information beyond baseline neurological assessment and may facilitate early individualized risk stratification using readily available clinical parameters.

## Introduction

Spinal cord injury (SCI) is a devastating form of central nervous system trauma associated with disastrous consequences and is frequently accompanied by severe vertebral fractures (1). Beyond the primary mechanical insult, the secondary pathophysiological cascade determines long-term neurological outcomes, among which the aberrant activation of the systemic immune–inflammatory response is particularly critical (2). Previous studies have indicated that abnormal immune activation not only exacerbates local secondary neural injury but can also induce systemic inflammatory response syndrome (SIRS), thereby hindering neural repair (3, 4). However, clinical trauma scenarios are often complex; severe vertebral fractures alone can induce intense stress responses. It remains unclear whether the “immune storm” observed in the acute phase of SCI stems primarily from the superposition of musculoskeletal soft tissue trauma or represents a pathological feature specific to CNS injury. Clarifying this distinction is crucial for establishing SCI-specific immune intervention targets.

In recent years, peripheral blood immune-inflammatory indices, such as the neutrophil-to-lymphocyte ratio (NLR) (5, 6), platelet-to-lymphocyte ratio (PLR) (7), and systemic immune-inflammation index (SII) (8), have been widely investigated as readily available biomarkers for prognostic assessment after traumatic injury and SCI (9). However, their reported prognostic performance remains inconsistent, partly because most previous studies evaluated individual biomarkers using conventional linear statistical approaches, which may not adequately capture the complex interactions and potential nonlinear relationships among inflammatory variables (10, 11). In addition, differences in injury patterns and trauma severity across study populations make direct comparison of immune-inflammatory profiles difficult and may contribute to heterogeneity among published findings. Therefore, there remains a need for robust and interpretable prediction strategies that integrate multidimensional clinical and laboratory information to improve early risk stratification in patients with SCI.

Based on these considerations, the present study had three objectives. First, we compared early peripheral immune-inflammatory profiles among patients with different traumatic injury patterns to provide clinical context for the inflammatory changes observed after SCI. Second, we evaluated the associations between routinely available early immune-inflammatory markers and 12-month neurological outcomes in patients with SCI. Third, we developed and internally validated an interpretable prediction model integrating clinical characteristics and early immune-inflammatory markers using conventional statistical methods together with machine learning techniques. Our primary objective was to establish a clinically applicable prediction model for early risk stratification, while the comparative analyses of different injury groups were intended to provide descriptive clinical context.

## Methods

### Study design and study population

This was a single-center retrospective cohort study approved by the Ethics Committee of the Affiliated Hospital of Zunyi Medical University (approval No. KLLY-2024-229). Consecutive trauma patients admitted to the emergency department, orthopedic department, or neurosurgery department between January 2022 and December 2024 were screened for eligibility.

The inclusion criteria were as follows: (1) acute traumatic injury requiring evaluation in the emergency, orthopedic, or neurosurgical departments; (2) age ≥14 years; (3) time from injury to hospital admission ≤24 h; (4) completion of the first hematological assessment within 1 h after admission; and (5) availability of complete clinical records and follow-up data. The presence or absence of spinal cord injury and vertebral fracture was subsequently determined according to computed tomography (CT) and/or magnetic resonance imaging (MRI) findings and was used for subgroup classification.

Patients were excluded if they met any of the following criteria: (1) concomitant traumatic brain injury; (2) abdominal visceral injury with massive hemorrhage or active infection; (3) severe extensive soft-tissue injury likely to cause substantial infection or immune alteration; (4) penetrating cervical injury; (5) pregnancy; (6) pre-existing severe hematologic disease, immune disorder, or malignancy; (7) receipt of immunomodulators, immunosuppressants, or systemic glucocorticoids at another hospital before admission; (8) history of severe liver disease, renal disease, or hypersplenism; (9) surgery or major trauma within 30 days before admission; (10) HIV infection or other clinically significant immunodeficiency; or (11) severe alcohol or substance abuse accompanied by acute withdrawal or intoxication.

### Grouping strategy and study framework

Patients were classified into four groups according to the presence or absence of SCI and vertebral fracture: (1) non-spinal injury (NSI), defined as acute trauma without SCI or vertebral fracture; (2) vertebral fracture (VF), defined as vertebral fracture without SCI; (3) isolated SCI (iSCI), defined as SCI without vertebral fracture; and (4) vertebral fracture combined with SCI (VF + iSCI), defined as concomitant vertebral fracture and SCI.

**Figure 1:**
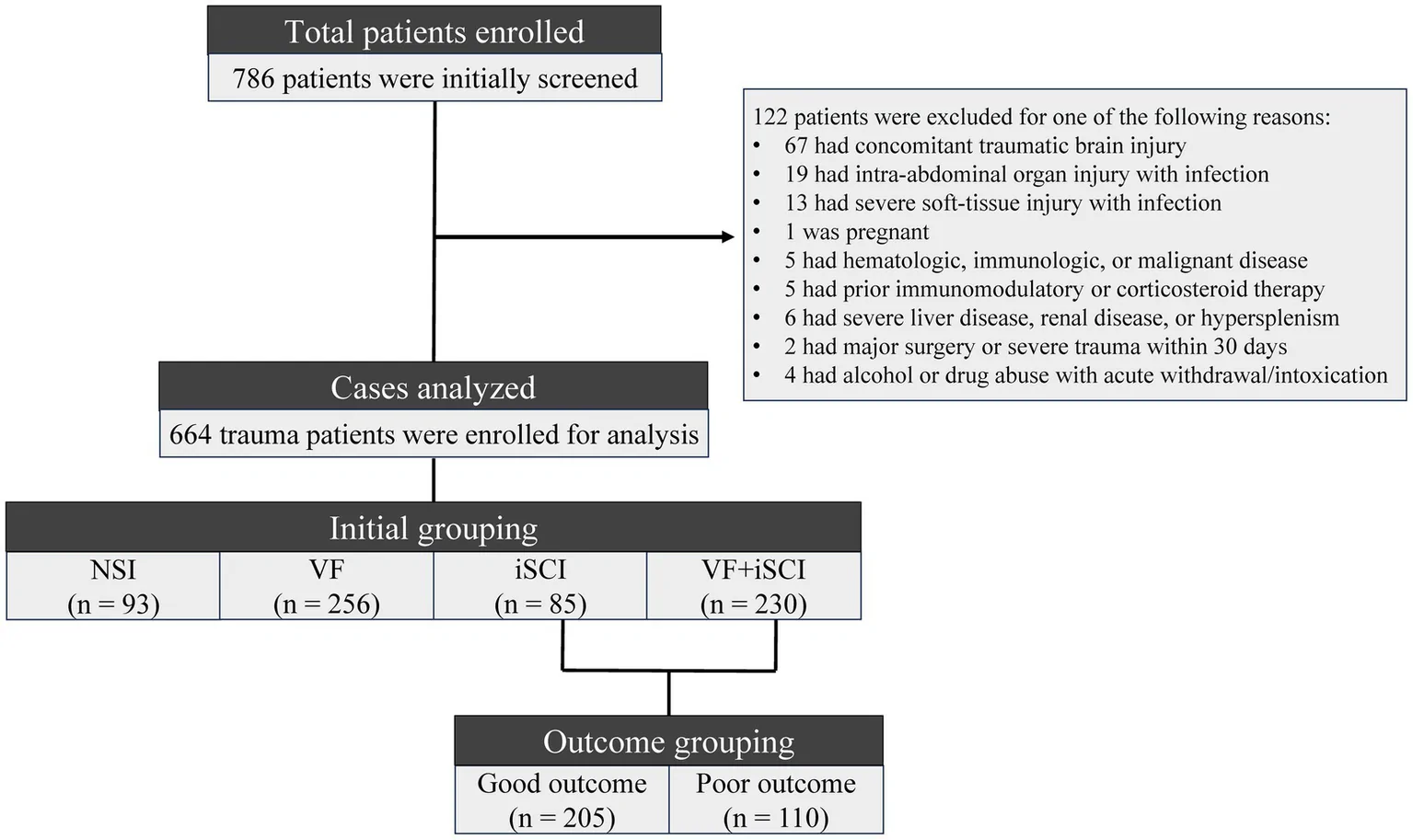
Flowchart of patient inclusion and study design.

For prognostic analyses, only patients with SCI were included. According to neurological recovery at the 12-month follow-up, these patients were further stratified into a favorable prognosis group (AIS grades D–E) and an unfavorable prognosis group (death or AIS grades A–C). This stratification was used to evaluate the prognostic value of early immune-inflammatory markers for long-term neurological outcomes (Figure 1).

### Data collection

Demographic and clinical data were collected for all eligible patients, including sex, age, blood type, mechanism of injury, SCI level, vertebral fracture level, Injury Severity Score (ISS), and AIS grade.

Laboratory parameters were obtained from the first peripheral blood sample collected after admission, including WBC, ANC, N%, absolute lymphocyte count (ALC), lymphocyte percentage (L%), absolute monocyte count (MON), monocyte percentage (M%), hemoglobin (HGB), and platelet count (PLT).

Coagulation parameters included prothrombin time (PT), activated partial thromboplastin time (APTT), thrombin time (TT), and fibrinogen (FIB). Clinical complications, including pneumonia (PNA) and deep vein thrombosis (DVT), were also recorded.

Based on these laboratory data, the following composite immune-inflammatory indices were calculated: NLR = NEU/LYM, PLR = PLT/LYM, monocyte-to-lymphocyte ratio (MLR = MON/LYM), systemic immune-inflammation index (SII = PLT × NEU/LYM), systemic inflammation response index (SIRI = NEU × MON/LYM), and aggregate index of systemic inflammation (AISI = PLT × NEU × MON/LYM).

Blood samples were collected during the early assessment phase after admission, generally before or during the initial stage of systemic resuscitation, such as large-volume fluid resuscitation or blood transfusion. According to the study protocol, the first hematological assessment was completed within 1 h after hospital admission. To characterize the timing of inflammatory marker measurement, the interval between injury and blood sampling was recorded for all patients. Neurological assessment was performed according to the International Standards for Neurological Classification of Spinal Cord Injury (ISNCSCI), and AIS grading was completed by neurosurgeons or spine surgeons who had received standardized training. Given the potential influence of spinal shock on very early neurological assessment, the AIS grade obtained at ≥72 h after admission was used as the baseline post-injury AIS grade in this study.

### Outcome assessment

The primary follow-up time point was 12 months after injury. Follow-up was conducted mainly through outpatient visits and supplemented by telephone interviews when necessary.

Whenever feasible, face-to-face neurological examination was prioritized, and neurological outcomes were assessed in accordance with ISNCSCI standards as much as possible to improve the reliability of outcome measurement. The primary outcome was neurological status at 1 year. A favorable outcome was defined as AIS grade D or E at follow-up, whereas an unfavorable outcome was defined as AIS grades A–C or death. Based on these outcome definitions, the present study aimed to investigate the prognostic relevance of early immune-inflammatory markers for long-term neurological recovery and the occurrence of clinical complications.

### Statistical analysis

Normality was assessed for all continuous variables before analysis. Normally distributed continuous variables are presented as mean ± standard deviation (SD), whereas non-normally distributed continuous variables are presented as median (interquartile range, IQR). Categorical variables are expressed as counts and percentages [n (%)]. For between-group comparisons, one-way analysis of variance (ANOVA) was used for continuous variables with normal distribution and homogeneity of variance; otherwise, the Kruskal–Wallis rank-sum test was applied. Categorical variables were compared using the Pearson chi-square test or Fisher’s exact test, as appropriate.

Prognostic analyses were restricted to patients with SCI. Clinical variables and immune-inflammatory markers were first entered as candidate predictors, and univariable logistic regression was performed to identify factors potentially associated with unfavorable outcomes. Given the substantial multicollinearity among several inflammatory indices, LASSO regression was subsequently used for variable selection. The optimal penalty parameter (λ) was determined by 10-fold cross-validation to reduce model complexity and improve the stability of feature selection. Variables retained after LASSO selection were then entered into a multivariable logistic regression model to estimate odds ratios (ORs) and 95% confidence intervals (CIs), thereby identifying independent predictors. Variance inflation factors (VIFs) were calculated to assess multicollinearity and ensure the robustness of model estimation.

To evaluate the incremental prognostic value of immune-inflammatory markers, a baseline model based on AIS alone and an integrated extended model incorporating immune-inflammatory indicators were constructed separately. ROC curves and the AUC were used to assess and compare the discriminatory performance of different models (12).

Based on the integrated extended feature set, both multivariable logistic regression and Extreme Gradient Boosting (XGBoost) were used for modeling (13), and their predictive performances were compared using ROC curves and AUC values. A nomogram was developed on the basis of the multivariable logistic regression model to facilitate individualized prediction of unfavorable outcomes. Calibration curves were used to evaluate the agreement between predicted probabilities and observed outcomes, while decision curve analysis (DCA) was performed to assess the clinical net benefit of the model across different threshold probabilities. For the XGBoost model, Shapley additive explanations (SHAP) were further applied to interpret model predictions and quantify the relative contribution of each variable (14).

Restricted cubic spline (RCS) analysis was performed to explore potential nonlinear associations between admission ANC and the risk of unfavorable 12-month outcomes. The median ANC value was used as the reference. The overall association and departure from linearity were assessed using likelihood ratio tests, with a two-sided *p* value <0.05 considered statistically significant.

All statistical tests were two-sided, and a *p* value <0.05 was considered statistically significant. Basic statistical analyses were performed using SPSS software (version 29.0; IBM Corp., Armonk, NY, USA), whereas advanced statistical analyses and model development were conducted using R software (version 4.4.1; R Foundation for Statistical Computing, Vienna, Austria).

## Results

### Baseline characteristics of patients with SCI

The baseline characteristics, injury patterns, and clinical outcomes of the 664 trauma patients are summarized in Table 1. Overall, there was no significant difference in sex distribution among the four groups (*p* = 0.070). Compared with patients without SCI (the NSI and VF groups), patients with SCI (the iSCI and VF + iSCI groups) were older, suggesting that advanced age may be associated with a higher likelihood of SCI after trauma. Analysis of injury mechanisms showed that falls from height were most frequent in the VF + iSCI group, indicating that high-energy vertical trauma may represent a major mechanism underlying vertebral fracture combined with SCI. No significant between-group difference was observed in the injury-to-blood sampling interval (*p* = 0.466).

**Table 1:** Baseline characteristics of 664 patients.

Variable	NSI (*n* = 93)	VF (*n* = 256)	iSCI (*n* = 85)	VF + iSCI (*n* = 230)	*p* value
Sex, male, *n* (%)	61 (65.6)	144 (56.3)	59 (69.4)	152 (66.1)	0.054^a^
Age, years	48.0 (37.0, 56.0)	53.0 (46.0, 60.0)^△^	58.0 (52.0, 65.5)^△*^	54(43.8, 62.0)^△#^	<0.001^c^
Injury-to-sampling time	8.00 (6.00, 12.00)	9.0 (6.00, 12.00)	10.0 (7.00, 15.00)	10.0 (7.00, 13.25)	0.466
Injury mechanism, *n* (%)					
Trip	29 (31.2)	86 (33.6)	30 (35.3)	57 (24.8)	0.105^a^
Fall from height	18 (19.4)	101 (39.5)^△^	45 (52.9)^△^	121 (52.6)^△*^	<0.001^a^
Motor vehicle injury	33 (35.5)	56 (21.9)	8 (9.4)^△^	36 (15.7)^△^	<0.001^a^
Animal attack injury	4 (4.3)	0 (0.0)^△^	0 (0.0)^△^	2 (0.9)	0.002^d^
Crush injury	9 (9.7)	13 (5.1)	2 (2.4)	14 (6.1)	0.192^a^
ISS, median [IQR]	9.0 (9.0, 13.0)	10.0 (9.0, 13.0)	20.0 (16.0, 25.5)^△*^	20.0 (13.0, 26.0)^△*^	<0.001^c^
AIS, *n* (%)					
Grade A	0 (0.0)	0 (0.0)	33 (38.8)^△*^	72 (31.3)^△*^	<0.001^d^
Grade B	0 (0.0)	0 (0.0)	10 (11.8)^△*^	22 (9.6)^△*^	<0.001^d^
Grade C	0 (0.0)	0 (0.0)	16 (18.8)^△*^	29 (12.6)^△*^	<0.001^d^
Grade D	0 (0.0)	0 (0.0)	26 (30.6)^△*^	106 (46.1)^△*^	<0.001^d^
Grade E	93 (100.0)	256 (100.0)	0 (0.0)^△*^	1 (0.4)^△*^	<0.001^d^
Spinal cord segment injury, *n* (%)					
Cervical segment	0 (0.0)	0 (0.0)	85 (100.0)^△*^	94 (40.9)^△*#^	<0.001^d^
Thoracic segment	0 (0.0)	0 (0.0)	0 (0.0)	31 (13.5)^△*#^	<0.001^d^
Lumbar segment	0 (0.0)	1 (0.4)	0 (0.0)	105 (45.7)^△*#^	<0.001^d^
Uninjured	93 (100.0)	255 (99.6)	0 (0.0)^△*^	0 (0.0)^△*^	<0.001^d^
Fractured segment, *n* (%)					
Cervical vertebrae	0 (0.0)	37 (14.5)^△^	0 (0.0)^*^	95 (41.3)^△*#^	<0.001^d^
Thoracic vertebrae	0 (0.0)	65 (25.4)^△#^	0 (0.0)	47 (20.4)^△#^	<0.001^d^
Lumbar vertebrae	0 (0.0)	154 (60.2)^△#^	0 (0.0)	88 (38.3)^△*#^	<0.001^d^
Non-fractured	93 (100.0)	0 (0.0)^△^	85 (100.0)^*^	0 (0.0)^#^	<0.001^d^
Surgical intervention, *n* (%)	28 (30.1)	202 (78.9)^△^	53 (62.4)^△*^	180 (78.3)^△#^	<0.001^a^
Time from injury to surgery, days	0.0 (0.0, 5.5)	3.0 (1.0, 5.0)^△^	3.0 (0.0, 5.0)^△^	3.0 (1.0, 5.0)^△^	<0.001^c^
WBC (10^9^ /L)	8.6 (6.6, 10.1)	8.2 (6.8, 9.6)	11.0 (9.1, 13.0)^△*^	11.2 (8.9, 14.5)^△*^	<0.001^c^
ANC (10^9^ /L)	6.8 (4.6, 8.1)	6.4 (5.1, 7.7)	9.4 (8.3, 11.1)^△*^	10.0 (7.7, 12.8)^△*^	<0.001^c^
*N* (%)	0.78 (0.71,0.82)	0.78 (0.73,0.83)	0.87 (0.81,0.91)^△*^	0.89 (0.86,0.92)^△*^	<0.001^c^
ALC (10^9^ /L)	1.2 (0.9, 1.5)	1.1 (0.8, 1.4)	1.0 (0.7, 1.1)^△*^	0.7 (0.5, 1.0)^△*#^	<0.001^c^
L (%)	0.14 (0.11, 0.18)	0.14 (0.10, 0.18)	0.08 (0.06, 0.12)^△*^	0.07 (0.05, 0.10)^△*#^	<0.001^c^
MON (10^9^ /L)	0.6 (0.5, 0.8)	0. 6 (0.4, 0.7) ^△^	0.5 (0.3, 0.7) ^△^	0.5 (0.3, 0.7) ^△^	<0.001^c^
M (%)	0.08 (0.06, 0.09)	0.07 (0.06, 0.08)	0.04 (0.03, 0.06)^△*^	0.04 (0.03, 0.06)^△*^	<0.001^c^
HGB (g/L)	121.6 ± 23.2	131.1 ± 19.3^△^	132.0 ± 15.4^△^	128.6 ± 20.2	<0.002^b^
PLT (10^9^ /L)	179.0 (142.5, 210.0)	178.0 (151.0, 217.0)	219.0 (179.0, 242.5)^△*^	179.0 (150.8, 219.50)^#^	<0.001^c^
PLR	151.2 (112.9, 205.8)	162.9 (121.0, 216.9)	244.3 (168.2, 311.7)^△*^	249.1 (179.4, 330.2)^△*^	<0.001^c^
MLR	0.53 (0.4, 0.74)	0.48 (0.35, 0.63)	0.44 (0.27, 0.63)	0.54 (0.37, 0.86) ^*#^	0.001^c^
SII	1045.9 (615.0, 1520.7)	1016.4 (660.4, 1527.7)	2175.0 (1466.4, 3199.1) ^△*^	2578.8 (1580.0, 3506.1) ^△*^	<0.001^c^
SIRI	3.8 (2.3, 5.3)	3.0 (1.9, 4.3)	4.2 (2.4, 7.2)^*^	5.7 (2.9, 9.8) ^△*^	<0.001^c^
AISI	610.9 (355.0, 1077.3)	519.3 (306.7, 839.7)	903.2 (450.8, 1344.9)^*^	957.4 (511.1, 1930.9) ^△*^	<0.001^c^
NLR	5.5 (3.8, 7.4)	5.6 (4.3, 8.2)	11.0 (6.8, 15.1) ^△*^	13.1 (8.8, 21.9) ^△*#^	<0.001^c^
APTT (Sec)	26.1 (23.9, 28.1)	26.4 (24.6, 29.1)	24.6 (23.6, 25.5)^△*^	25.5 (24.0, 27.7)^*#^	<0.001^c^
PT (Sec)	10.8 (10.3, 11.7)	10.8 (10.3, 11.7)	10.2 (9.8, 10.6)^△*^	10.6 (9.9, 11.6)^#^	<0.001^c^
TT (Sec)	17.1 (16.1, 18.2)	17.1 (16.3, 18.0)	17.6 (17.0, 18.2)^*^	17.4 (16.5, 18.3)	0.004^c^
FIB	2.5 (2.0, 3.3)^*^	3.1 (2.4, 3.7)	2.5 (1.9, 3.0)^*^	2.6 (2.1, 3.2)^*^	<0.001^c^
PNA, *n* (%)	13 (14.0)	73 (28.5)^△^	35 (41.2)^△^	95 (41.3)^△*^	<0.001^a^
DVT, *n* (%)	1 (1.1)	13 (5.1)	13 (15.3)^△*^	28 (12.2)^△*^	<0.001^a^
Prognosis, *n* (%)					<0.001^d^
Death	0 (0.0)	0 (0.0)	17 (20.0)^△*^	24 (10.4)^△*^	
No improvement	0 (0.0)	0 (0.0)	0 (0.0)	20 (8.7)^△*#^	
Symptoms improve	0 (0.0)	0 (0.0)	68 (80.0)^△*^	186 (80.9)^△*^	
Normal	93 (100.0)	256 (100.0)	0 (0.0)^△*^	0 (0.0)^△*^	

*p* < 0.05 was considered statistically significant. Symbols indicate group comparisons: △, compared with the non-spinal injury group (*p* < 0.05); *, compared with the fracture group (*p* < 0.05); #, compared with the spinal cord injury (SCI) group (*p* < 0.05). Data are presented as mean ± SD, *n* (%), or median. Intergroup comparisons were performed based on data types.

a Chi-square test.

b One-way analysis of variance (ANOVA).

c Kruskal–Wallis test.

d Fisher’s exact test.

AISI, aggregate index of systemic inflammation; AIS, abbreviated injury scale; ANC, absolute neutrophil count; APTT, activated partial thromboplastin time; DVT, deep vein thrombosis; FIB, fibrinogen; HGB, hemoglobin; LYM, lymphocyte count; M, monocyte count; MLR, monocyte-to-lymphocyte ratio; NLR, neutrophil-to-lymphocyte ratio; PLR, platelet-to-lymphocyte ratio; PLT, platelet count; PT, prothrombin time; SII, systemic immune-inflammation index; SIRI, systemic inflammation response index; TT, thrombin time; WBC, white blood cell count.

**Figure 2:**
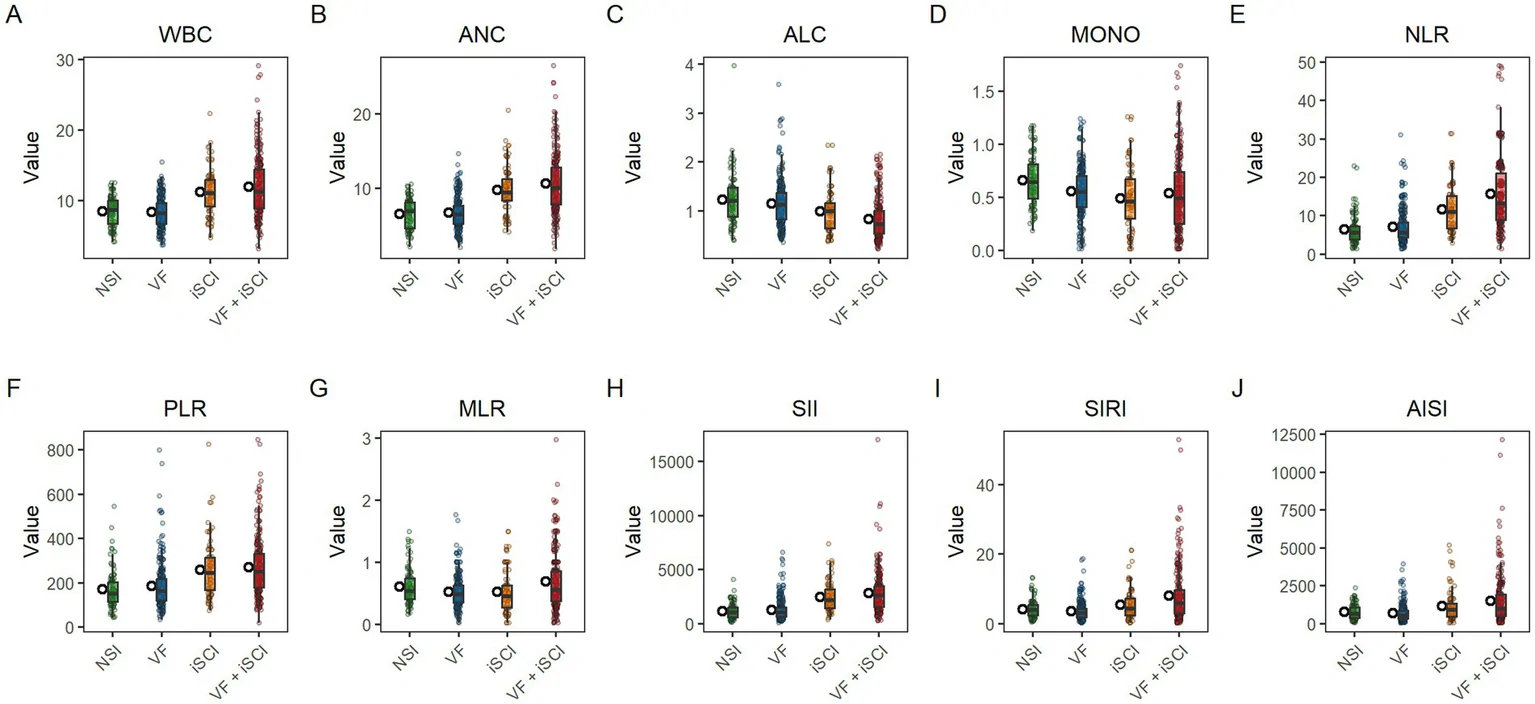
Comparison of peripheral blood immune–inflammatory markers across different injury patterns. The box plots illustrate the distribution of admission immune indices among the NSI, VF, iSCI, and VF + iSCI groups. Panels display: **(A)** WBC; **(B)** ANC; **(C)** ALC; **(D)** MONO; **(E)** NLR; **(F)** PLR; **(G)** MLR; **(H)** SII; **(I)** SIRI; **(J)** AISI. The horizontal line within each box represents the median, the white circle denotes the mean, and the box spans the IQR. Scattered points represent individual patient values.

Regarding the distribution of injury levels, nearly all patients in the iSCI group had cervical cord injury, whereas the VF + iSCI group showed a more multilevel distribution, suggesting greater anatomical complexity in patients with SCI accompanied by vertebral fracture. Laboratory findings demonstrated more pronounced systemic immune-inflammatory activation in patients with SCI. Compared with the non-SCI groups, the iSCI and VF + iSCI groups had significantly higher WBC, ANC, and N%, but significantly lower ALC and L%. Accordingly, NLR, PLR, SII, and SIRI were all markedly elevated in both SCI groups relative to the non-SCI groups (all *p* < 0.001). These differences were visually illustrated by the raincloud plots of immune parameters in Figure 2, suggesting that acute SCI induces a systemic inflammatory response characterized by neutrophil predominance.

Further analysis of complications and clinical outcomes showed that the incidences of PNA and DVT were significantly higher in the iSCI and VF + iSCI groups than in the non-SCI groups (both *p* < 0.001). Taken together, Table 1 and Figure 2 indicate that, compared with isolated soft-tissue injury or isolated vertebral fracture, SCI is associated with greater injury burden and more profound systemic immune-inflammatory dysregulation, suggesting that SCI may be an important driver of post-traumatic immune disturbance.

### Clinical and injury characteristics of patients with unfavorable outcomes

At the 12-month follow-up endpoint, a total of 664 patients achieved a good outcome (AIS grades D–E), while 110 experienced a poor outcome (AIS grades A–C or death) (Table 2). Compared to the Good Outcome group, patients in the Poor Outcome group exhibited a significantly higher propensity for advanced age and male sex (*p* < 0.05) and were more frequently injured by falls from height. Regarding injury severity, the Poor Outcome group was characterized by higher ISS scores, severe neurological deficits predominating in AIS grades A and B, and high-level (cervical) spinal cord injuries (*p* < 0.001). Laboratory investigations revealed a state of heightened inflammatory activation in the Poor Outcome group, manifested by significantly elevated neutrophil-related parameters and marked suppression of lymphocytes. Correspondingly, all composite immune–inflammatory indices (NLR, PLR, SII, SIRI, and AISI) were significantly elevated in the Poor Outcome group (*p* < 0.001). Furthermore, the Poor Outcome group exhibited more severe coagulation disturbances, and the incidence of PNA and DVT was significantly higher compared to the Good Outcome group (*p* < 0.01).

**Table 2:** Comparison between patients with favorable and poor 1-year outcomes after spinal cord injury.

Variable	Good outcome (*n* = 205)	Poor outcome (*n* = 110)	*p* value
Sex, Male, *n* (%)	132 (64.4)	79 (71.8)	0.181^a^
Age, years	55.0 (45.0, 61.0)	58.0 (48.0, 68.3)	0.015^c^
Injury-to-sampling time	10.00 (7.00, 13.00)	10.00 (7.00, 16.00)	0.408
Injury mechanism, *n* (%)			
Trip	51 (24.9)	36 (32.7)	0.137^a^
Fall from height	107 (52.2)	58 (52.7)	0.928^a^
Motor vehicle injury	35 (17.1)	9 (8.2)	0.030^a^
Animal attack injury	2 (1.0)	0 (0.0)	0.544^d^
Crush injury	10 (4.9)	6 (5.5)	0.824^a^
ISS, median [IQR]	16.0 (10.0, 21.0)	26.0 (25.0, 30.0)	<0.001^c^
AIS, *n* (%)			
Grade A	13 (6.3)	92 (83.6)	<0.001^a^
Grade B	15 (7.3)	18 (16.4)	0.012^a^
Grade C	47 (22.9)	0 (0.0)	<0.001^a^
Grade D	130 (63.4)	0 (0.0)	<0.001^a^
Spinal cord segment injury, *n* (%)			
Cervical segment	99 (48.3)	80 (72.7)	<0.001^a^
Thoracic segment	18 (8.8)	13 (11.8)	0.388^a^
Lumbar segment	88 (42.9)	17 (15.5)	<0.001^a^
Fractured segment, *n* (%)			
Cervical vertebrae	53 (25.9)	42 (38.2)	0.023^a^
Thoracic vertebrae	29 (14.1)	18 (16.4)	0.599^a^
Lumbar vertebrae	76 (37.1)	12 (10.9)	<0.001^a^
Non-fractured	47 (22.9)	38 (34.5)	0.027^a^
Surgical intervention, *n* (%)	151 (73.7)	82 (74.5)	0.864^a^
Time from injury to surgery, days	3.0 (0.0, 5.0)	3.0 (0.0, 5.0)	0.823^c^
WBC (10^9^ /L)	10.4 (8.5, 13.0)	12.5 (10.1, 15.3)	<0.001^c^
ANC (10^9^ /L)	9.3 (7.3, 11.8)	11.0 (9.0, 13.5)	<0.001^c^
*N* (%)	0.88 (0.83,0.92)	0.89 (0.87,0.92)	0.140^c^
ALC (10^9^ /L)	0.8 (0.6, 1.1)	0.8(0.6, 1.1)	0.606^c^
L (%)	0.07 (0.05, 0.11)	0.07 (0.05, 0.09)	0.057^c^
MON (10^9^ /L)	0.4 (0.2, 0.7)	0.5 (0.3, 0.8)	0.052^c^
M (%)	0.04 (0.02, 0.06)	0.04 (0.03, 0.06)	0.548^c^
HGB (g/L)	131.5 ± 19.7	125.8 ± 19.1	0.011^b^
PLT (10^9^ /L)	196.0 (161.0,227.0)	178.0 (148.0,230.0)	0.140^c^
PLR	262.8 (181.6, 330.9)	228.8 (168.7, 316.8)	0.127^c^
MLR	0.50 (0.32, 0.80)	0.57 (0.41, 1.00)	0.069^c^
SII	2298.1 (1477.5, 3484.6)	2516.3 (1695.7, 3421.2)	0.251^c^
SIRI	4.3 (2.6, 7.9)	7.0 (2.9, 12.6)	0.002^c^
AISI	863.9 (471.7, 1530.6)	1117.0 (560.9, 2288.6)	0.008^c^
NLR	12.4 (7.7, 18.3)	12.8 (10.1, 18.9)	0.092^c^
APTT (Sec)	25.4 (24.2,27.6)	24.7 (23.5, 26.5)	0.002^c^
PT (Sec)	10.5 (9.9, 11.4)	10.5 (9.9, 11.2)	0.378^c^
TT (Sec)	17.3 (16.5, 18.1)	17.7 (16.8, 18.4)	0.026^c^
FIB	2.8 (2.2, 3.3)	2.5 (1.9, 2.9)	<0.001^c^
PNA, *n* (%)	76 (37.1)	54 (49.1)	0.039
DVT, *n* (%)	21 (10.2)	20 (18.2)	0.046

a Chi-square test.

b Student’s *t*-test.

c Mann–Whitney U test.

d Fisher’s exact test.

### Associations among immune-inflammatory markers, complications, and outcomes

**Figure 3:**
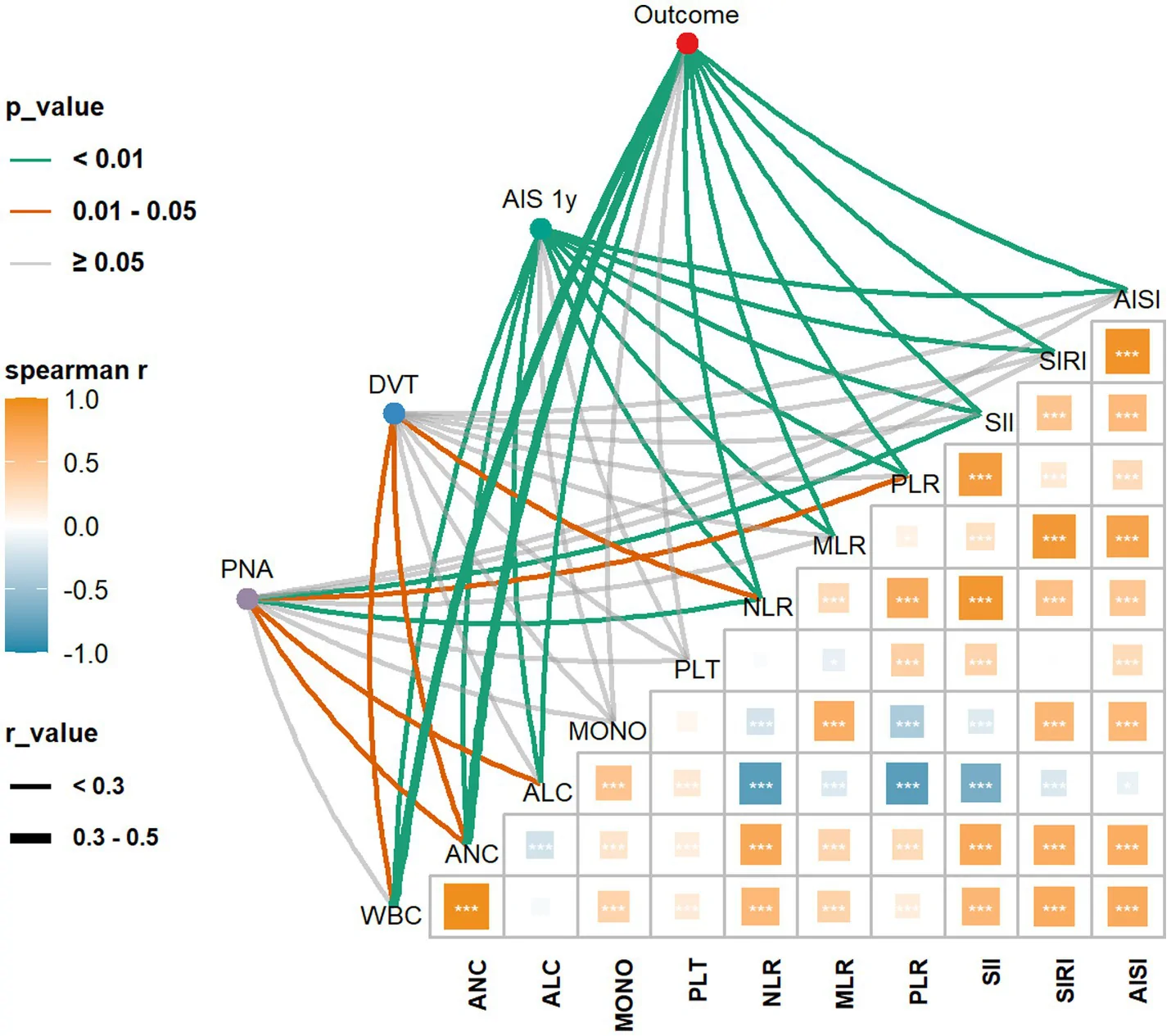
Correlation network analysis of clinical outcomes, complications, and immune-related parameters in patients with spinal cord injury. Nodes represent clinical outcomes (Outcome), 1-year AIS score (AIS 1y), complications (DVT, PNA), and immune-related parameters (WBC, ANC, ALC, MONO, PLT, NLR, MLR, PLR, SII, SIRI, AISI). Edges indicate Spearman correlations, with color denoting significance (green: *p* < 0.01; orange: 0.01–0.05; gray: *p* ≥ 0.05) and line thickness reflecting correlation strength (thicker: 0.3 ≤ |*r*| < 0.5; thinner: |*r*| < 0.3). The heatmap shows pairwise correlation coefficients (*r*), with red for positive and blue for negative correlations; “***” indicates *p* < 0.001.

Correlation network analysis further delineated the interrelationships among immune-inflammatory status, complications, and clinical outcomes in patients with SCI. As shown in the heatmap in Figure 3, most immune-inflammatory parameters were strongly positively correlated with one another, suggesting that these indices may reflect a common background of inflammatory activation after acute SCI. Outcome status, 12-month AIS grade, PNA, and DVT were all associated with multiple immune-inflammatory markers. Among them, WBC, ANC, and NLR showed particularly strong associations with unfavorable outcomes. At the same time, PNA and DVT were also significantly correlated with several immune-inflammatory indices, indicating that acute-phase systemic immune-inflammatory activation may be related not only to poor neurological recovery but also to the development of secondary complications.

### Risk factors for unfavorable prognosis in patients with SCI

The results of univariable logistic regression analysis are summarized in Supplementary Table 1. Overall, baseline neurological injury severity emerged as the strongest correlate of prognosis, with complete injuries showing the greatest association with unfavorable outcomes. Greater overall injury burden and cervical cord involvement were likewise associated with an increased risk of poor outcome.

Among laboratory parameters, elevated inflammation-related markers, particularly WBC and ANC, were associated with unfavorable outcomes. Several composite inflammatory indices also showed significant associations. In addition, coagulation abnormalities and in-hospital complications, including PNA and DVT, were related to adverse outcomes.

Taken together, the univariable analysis suggested that baseline neurological severity, injury characteristics, systemic inflammatory activation, coagulation disturbances, and secondary complications may all contribute to long-term outcome heterogeneity in patients with SCI.

### LASSO regression identified key predictors

**Figure 4:**
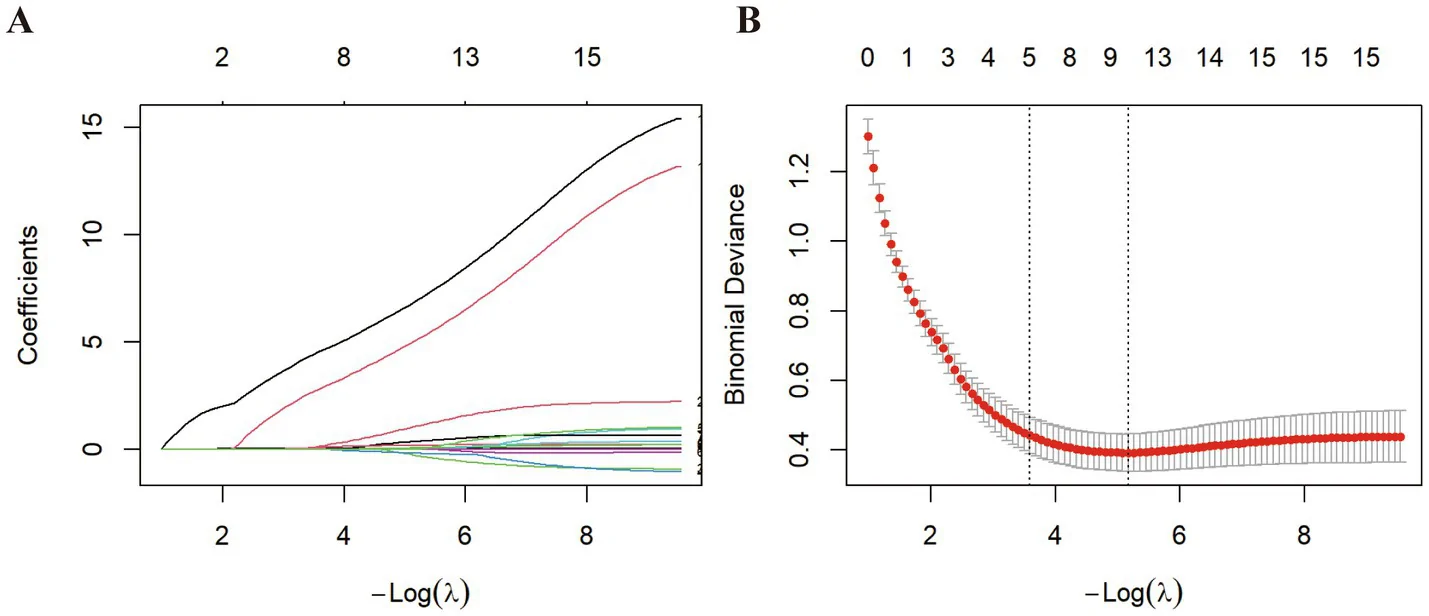
LASSO regression for variable selection. **(A)** LASSO coefficient profiles of candidate variables. Coefficients are plotted against −log(*λ*); each curve represents one variable. Vertical dashed lines indicate *λ*_min_ and *λ*_1SE_. **(B)** Ten-fold cross-validated binomial deviance. The red line represents the mean deviance, and the shaded area indicates ± 1 standard error. Vertical dashed lines denote *λ*_min_ (minimum deviance) and *λ*_1SE_ (minimum deviance plus one standard error).

To identify the predictors most strongly associated with unfavorable neurological outcomes from multidimensional candidate variables, LASSO regression was performed following the univariable analysis. Ten-fold cross-validation was used, with binomial deviance as the performance metric, to achieve dimensionality reduction and minimize overfitting. At the optimal regularization parameter (λ_min), nine candidate variables with non-zero coefficients were retained. Under the more parsimonious λ_1se criterion, the model was further shrunk, and five key predictors were ultimately selected: ISS, AIS grade A, AIS grade B, cervical cord involvement, and ANC (Figure 4).

VIFs were then used to assess multicollinearity in the model. The results showed substantial collinearity between AIS grade A and AIS grade B, together with quasi-complete separation. After considering statistical stability and clinical interpretability, AIS grade B was excluded from the final model. Accordingly, four core variables—ISS, AIS grade A, cervical cord involvement, and ANC—were retained for subsequent multivariable logistic regression and machine learning modeling (see Table 3).

**Table 3:** Multivariable logistic regression model for predicting poor 1-year outcomes after.

Variables	Multivariable analysis		
	OR	95% CI	*p* value
ISS, median [IQR]	1.184	1.000–1.288	<0.001
AIS A	25.972	10.668–69.423	<0.001
Cervical segment	4.325	1.711–11.748	0.002
ANC (10^9^ /L)	1.249	1.101–1.435	<0.001

### Predictive performance and incremental value of the model

**Figure 5:**
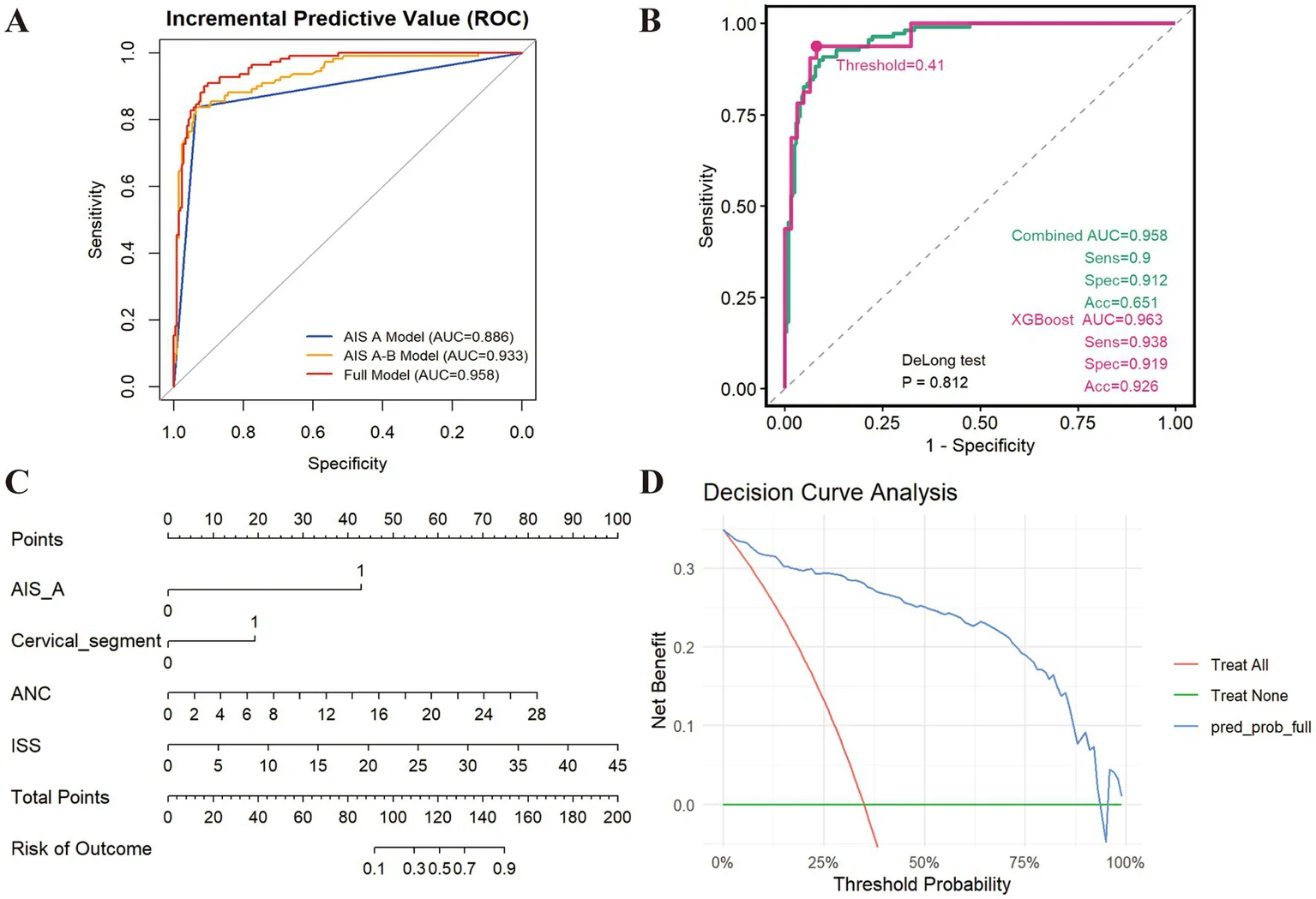
Predictive performance and clinical utility of the model. **(A)** ROC curves comparing the prognostic performance of baseline AIS grade A alone, the intermediate model incorporating AIS grades A and B, and the full multivariable logistic regression model incorporating four core predictors. **(B)** ROC curve of the XGBoost model. **(C)** Nomogram for predicting the probability of unfavorable 1-year outcomes based on ISS, AIS grade A, cervical cord involvement, and ANC. **(D)** Decision curve analysis showing the net clinical benefit of the model across a range of threshold probabilities.

Baseline AIS grade A alone showed strong prognostic value, with an AUC of 0.886. The addition of AIS grade B yielded an intermediate model with an AUC of 0.933. The full multivariable logistic regression model incorporating the four core predictors achieved further improvement in discriminative performance, with an AUC of 0.958, a sensitivity of 0.900, and a specificity of 0.912, significantly outperforming the baseline model (DeLong test, *p* < 0.001) (Figure 5A).

### Internal validation and calibration of the model

Internal validation of the extended logistic regression model was performed using bootstrap resampling (*B* = 1,000). The results demonstrated good model stability and high discriminative ability. The optimism-corrected C-statistic was 0.955, representing only a minimal decrease of 0.003 from the apparent performance (0.958), indicating limited overfitting. In addition, the model showed good explanatory performance, with an optimism-corrected Nagelkerke *R*^2^ of 0.746. Calibration analysis showed that the corrected calibration slope was 0.953, the intercept was −0.017, and the maximum absolute error (Emax) was 1.3%, indicating close agreement between predicted probabilities and observed outcomes (Supplementary Figure 1). Overall, the model demonstrated not only excellent discrimination but also good calibration.

### Comparison with the machine learning model

To further explore potential nonlinear relationships and interactions among variables, an XGBoost model was constructed using the same feature set. The XGBoost model achieved an AUC of 0.963, with a sensitivity of 0.938 and a specificity of 0.919 at the optimal cutoff. Overall, the XGBoost model also showed high predictive performance, although its AUC was slightly lower than that of the multivariable logistic regression model (Figure 5B).

### Construction of a clinical tool and decision curve analysis (DCA)

To facilitate individualized clinical risk assessment, a nomogram was developed based on the multivariable logistic regression model. The nomogram assigned corresponding point values to ISS, AIS grade A, cervical cord involvement, and ANC, and the total score was translated into the predicted probability of unfavorable outcome (Figure 5C). Within the nomogram, AIS grade A contributed the greatest weight, followed by cervical cord involvement, whereas ISS and ANC provided additional risk stratification as continuous variables.

DCA showed that the model yielded favorable clinical net benefit across a threshold probability range of 0 to 90%. In particular, when the threshold probability exceeded 35%, a strategy based on model-guided risk stratification and intervention was clearly superior to the “treat-all” or “treat-none” strategies (Figure 5D). These findings indicate that, in addition to strong statistical performance, the model also has potential clinical utility.

### Model interpretability analysis based on SHAP

**Figure 6:**
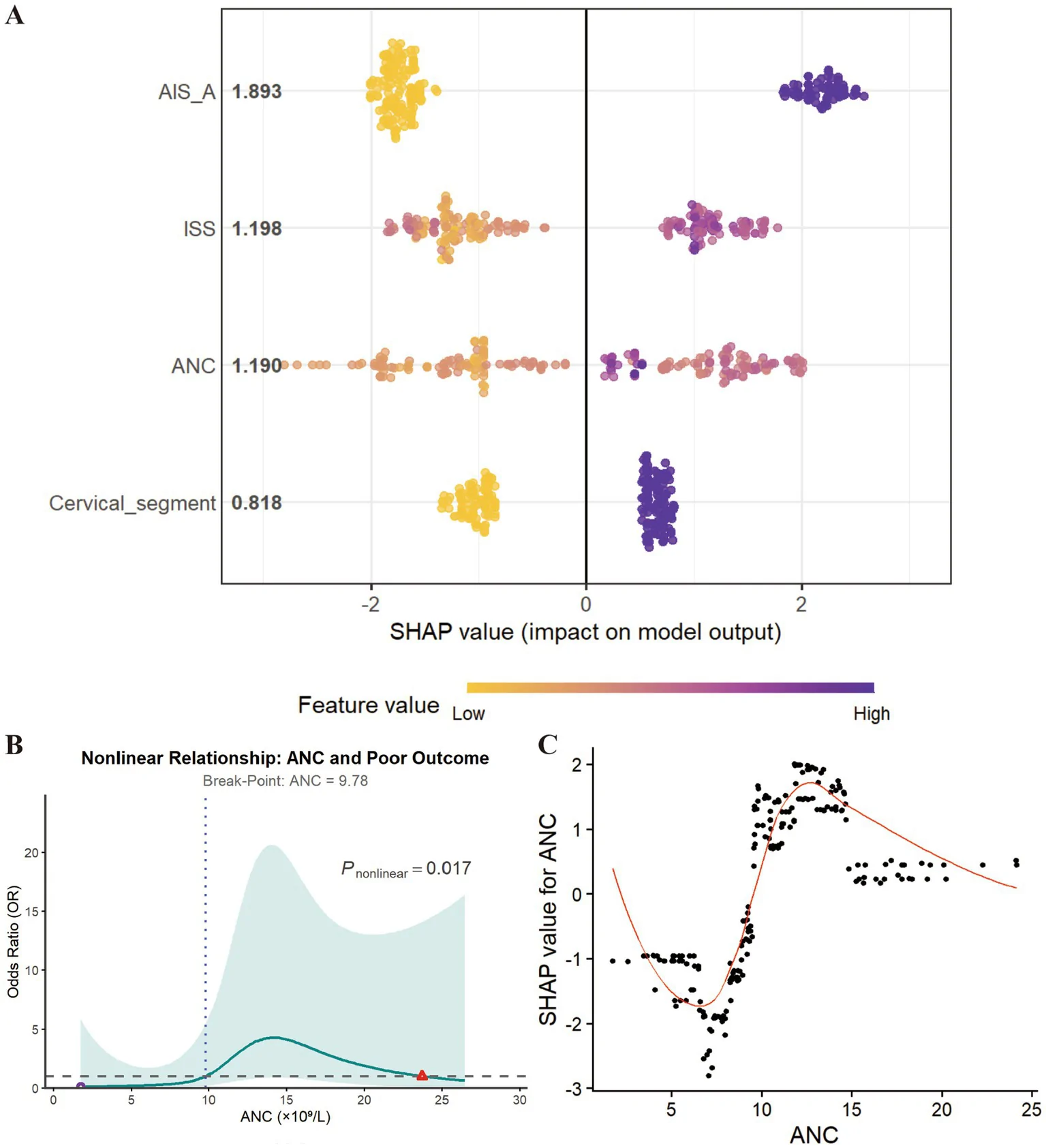
SHAP-based interpretation of the XGBoost model. **(A)** Feature importance ranked by mean absolute SHAP values. **(B)** RCS analysis demonstrating a nonlinear association between admission ANC and the risk of unfavorable neurological outcomes at 12 months. **(C)** SHAP dependence plot for ANC.

To improve the transparency and clinical interpretability of the XGBoost model, SHapley Additive exPlanations (SHAP) were used to quantify the contribution of each feature to model predictions. Ranking features according to the mean absolute SHAP value showed that the relative contributions of the variables, in descending order, were AIS grade A, ISS, ANC, and cervical cord involvement (Figure 6A). Among these, AIS grade A had the greatest predictive contribution at the individual level, whereas ISS and ANC provided important continuous risk information.

To further characterize the potential nonlinear association between ANC and unfavorable outcomes, RCS analysis was performed. The analysis demonstrated a significant nonlinear association between admission ANC and the risk of unfavorable outcomes (*P* for nonlinearity = 0.017). The estimated association was relatively flat at lower ANC levels but became steeper above an inflection point of 9.78 × 10^9^/L, suggesting a potential threshold effect of ANC on unfavorable neurological outcomes (Figure 6B). Similarly, ANC showed a nonlinear relationship with model-predicted risk, rather than a simple linear increase (Figure 6C). These findings suggest that machine learning models may identify complex risk patterns that are difficult to fully capture using conventional linear models.

## Discussion

In this study, we systematically characterized the early systemic immune–inflammatory response after traumatic spinal cord injury (SCI) and evaluated its association with 12-month unfavorable outcomes by integrating conventional statistical analyses with interpretable machine learning approaches. Compared with patients without SCI, patients with SCI exhibited more pronounced systemic inflammatory activation, characterized by increased WBC, ANC, and N%, reduced lymphocyte counts, and marked elevations in NLR, PLR, SII, and SIRI. These findings suggest that SCI is associated with a systemic immune–inflammatory response that extends beyond the local consequences of spinal trauma.

This is consistent with previous evidence that SCI possesses immunopathological features distinct from trauma involving other body regions (15) and with the concept of SCI-induced immune dysregulation syndrome (SCI-IDS) (16, 17). Acute SCI can rapidly alter catecholamine and glucocorticoid levels through sympathetic nervous system overactivation and hypothalamic–pituitary–adrenal (HPA) axis dysfunction (18, 19), thereby reshaping the quantitative distribution and functional state of peripheral immune cells (20, 21). In our cohort, the significantly higher incidences of pneumonia and deep vein thrombosis among patients with SCI further support the presence of systemic consequences associated with this neuroimmune dysregulation and may reflect the complex interaction between inflammation, coagulation, and autonomic dysfunction after SCI (22). Importantly, although multiple inflammatory indices were significantly elevated in patients with SCI and unfavorable outcomes, ANC was the inflammatory marker ultimately retained in the final prognostic model, suggesting that it may provide additional prognostic information beyond neurological and overall injury severity.

Among the inflammatory parameters evaluated, ANC showed the most consistent prognostic relevance. Elevated ANC was associated with unfavorable outcomes in univariable analysis and was subsequently retained by LASSO regression as one of the key predictors. After accounting for multicollinearity, the final model incorporated AIS grade A, ISS, cervical cord involvement, and ANC. The biological plausibility of this association is supported by the established role of neutrophils in acute tissue injury and secondary inflammatory responses (23). Activated neutrophils can release reactive oxygen species, proteases, and neutrophil extracellular traps, potentially contributing to blood–spinal cord barrier disruption and secondary tissue damage (24, 25). Nevertheless, circulating ANC should be regarded primarily as an accessible marker of the systemic inflammatory state rather than direct evidence of neutrophil-mediated neurological injury.

Importantly, the association between ANC and unfavorable outcomes was not simply linear. RCS analysis demonstrated a significant nonlinear association (P for nonlinearity = 0.017), with a steeper increase in estimated risk above approximately 9.78 × 10^9^/L. This finding suggests a potential threshold effect and indicates that markedly elevated ANC may identify patients with a particularly high-risk inflammatory profile (26). However, the identified threshold should be considered exploratory and requires external validation before clinical application. In contrast, although SIRI and other composite inflammatory indices were significantly elevated in SCI patients and in those with unfavorable outcomes, they were not retained in the final predictive model. Therefore, these indices may reflect the overall inflammatory state but should not be interpreted as independent prognostic factors based on the present analysis.

Neurological injury severity remained the dominant determinant of long-term outcome in our cohort (27). Concurrently, the strong association between cervical spinal cord injury and poor outcomes reflects not only more severe motor and sensory deficits but also potentially widespread impairment of autonomic nervous system regulation, which can further amplify immune and endocrine disorders. This is consistent with clinical observations of higher infection and mortality rates in patients with high-level SCI (28). SHAP analysis further improved the interpretability of the predictive model (29), showing that AIS grade A contributed most to the model predictions, followed by ISS, ANC, and cervical cord involvement. Importantly, the final logistic regression model, which integrated neurological severity, overall injury burden, anatomical injury characteristics, and systemic inflammation, achieved an AUC of 0.958, with good internal calibration and optimism-corrected discrimination. The XGBoost model achieved a comparable AUC of 0.963, with a higher sensitivity of 0.938, and provided a complementary approach for exploring potential nonlinear relationships and interactions among predictors (30). These findings suggest that, although neurological severity remains the fundamental determinant of prognosis, incorporating peripheral inflammatory information, particularly ANC, may provide additional prognostic information and improve early risk stratification of patients with SCI.

Overall, our findings support a multidimensional approach to early prognostic assessment after SCI. Neurological severity provides the primary prognostic information, whereas ISS, anatomical injury characteristics, and peripheral inflammatory markers may provide additional risk stratification. Because ANC is routinely available, inexpensive, and rapidly obtainable, its incorporation into clinical prediction models may improve early identification of patients at increased risk of unfavorable outcomes. Nevertheless, the present model should be regarded as an internally validated research tool until its performance and clinical utility are confirmed in independent cohorts.

This study also has certain limitations. First, as a single-center retrospective study, selection bias may exist. Second, the inflammatory assessment was based primarily on a single early peripheral blood measurement and therefore could not capture the dynamic evolution of the systemic immune response. Third, the primary unfavorable outcome combined death with AIS grades A–C. Although clinically useful for defining a high-risk group, death and neurological non-recovery are not equivalent outcomes; future studies should analyze mortality and neurological recovery separately and consider longitudinal AIS grade conversion.

## Conclusion

In conclusion, acute SCI was associated with a more pronounced systemic immune–inflammatory response than trauma without SCI, characterized predominantly by neutrophil activation and lymphocyte suppression. Among the inflammatory parameters evaluated, ANC provided the most consistent incremental prognostic information and was retained in the final prediction model together with neurological severity, cervical cord involvement, and overall injury burden. Importantly, the association between ANC and unfavorable outcome was nonlinear, with a more pronounced increase in estimated risk above approximately 9.78 × 10^9^/L. These findings support the incorporation of routinely available inflammatory markers, particularly ANC, as adjunctive information rather than replacements for standardized neurological assessment. Further prospective and externally validated studies are needed to establish the generalizability, clinical utility, and biological mechanisms underlying these associations.

## Transparency, rigor and reproducibility summary

The study was not formally pre-registered because it is a retrospective observational study based on existing clinical data. The analysis plan was not formally pre-registered; however, the statistical analysis strategy, including the use of restricted cubic splines (RCS) and machine learning algorithms (XGBoost), was pre-specified by the research team prior to data analysis. A formal sample size calculation was not performed *a priori*; the sample size was determined by the availability of eligible cases in the trauma database of the Affiliated Hospital of Zunyi Medical University during the study period. However, the final sample size of 110 participants exceeded the minimum requirement of 50 subjects calculated to be necessary for the multivariate analysis. The number of participants screened, excluded, and analyzed is documented in the study flow diagram to ensure accounting for all experimental subjects. Due to the retrospective nature of data extraction, blinding of data collectors to clinical outcomes was not implemented; however, bias was minimized by utilizing objective laboratory markers and established neurological standards (AIS grade). De-identified data from this study are available from the corresponding author upon reasonable request. Analytic code used to conduct the statistical analyses is available from the corresponding author upon request. The authors agree to provide the full content of the manuscript on request by contacting the corresponding author.

## Data Availability

The raw data supporting the conclusions of this article will be made available by the authors, without undue reservation.
