# Supplementary material for: An interpretable machine learning model integrating early immune biomarkers for predicting outcomes after spinal cord injury
Source: Front Neurol. 2026 Sep 8;17:1855360. doi: 10.3389/fneur.2026.1855360 (PMC13593449; doi:10.3389/fneur.2026.1855360)
Supplement: Supplementary file 1 [file Supplementary_file_1.docx]

**SUPPLEMENTARY DATA**

**Supplementary TABLE 1** Univariate logistic regression analysis of factors associated with poor 1-year outcomes after spinal cord injury.

| Variables | Univariable analysis | | |
| --- | --- | --- | --- |
|  | OR | 95% CI | *P* Value |
| Motor vehicle injury | 0.433 | 0.200-0.937 | 0.034 |
| ISS, median [IQR] | 1.321 | 1.240-1.408 | ＜0.001 |
| AIS A | 75.487 | 35.465-160.676 | ＜0.001 |
| AIS B | 2.478 | 1.195-5.138 | 0.015 |
| Cervical segment | 2.855 | 1.730-4.713 | ＜0.001 |
| Lumbar segment | 0.243 | 0.135-0.437 | ＜0.001 |
| Cervical vertebrae | 1.771 | 1.079-2.908 | 0.024 |
| Lumbar Vertebrae | 0.208 | 0.107-0.403 | ＜0.001 |
| FIB | 0.637 | 0.473-0.858 | 0.003 |
| APTT (Sec) | 0.903 | 0.842-0.968 | 0.004 |
| WBC (10^9^ /L) | 1.140 | 1.072-1.212 | ＜0.001 |
| ANC (10^9^ /L) | 1.149 | 1.077-1.227 | ＜0.001 |
| HGB | 0.984 | 0.972-0.997 | 0.012 |
| SIRI | 1.050 | 1.016-1.086 | 0.004 |
| AISI | 1.000 | 1.000-1.000 | 0.010 |
| PNA, n (%) | 1.637 | 1.024-2.616 | 0.040 |
| DVT, n (%) | 1.947 | 1.004-3.776 | 0.049 |

Notes: OR = Odds Ratio; CI = Confidence Interval.

**Supplementary TABLE 2** Sensitivity analysis of the incremental predictive value after excluding death from the composite outcome.

| **Analysis** | ***n*** | **Events** | **AIS A model AUC** | **Full model AUC** | **ΔAUC** | **Optimism-corrected C-statistic** |
| --- | --- | --- | --- | --- | --- | --- |
| Composite outcome (death or AIS A–C) | 315 | 110 | 0.886 | 0.958 | 0.072 | 0.955 |
| Survivor-only outcome (AIS A–C) | 274 | 69 | 0.874 | 0.948 | 0.074 | 0.944 |

**Supplementary Fig.1** Calibration of the extended logistic regression model after bootstrap internal validation

Calibration curve of the model after bootstrap resampling (B = 1000). The optimism-corrected curve shows good agreement between predicted and observed outcomes (slope = 0.953, intercept = −0.017; Emax = 1.3%), indicating good calibration with minimal overfitting (corrected C-statistic = 0.955).
