# Supplementary material for: An interpretable machine learning model integrating early immune biomarkers for predicting outcomes after spinal cord injury
Source: Front Neurol. 2026 Sep 8;17:1855360. doi: 10.3389/fneur.2026.1855360 (PMC13593449; doi:10.3389/fneur.2026.1855360)
Supplement: Supplementary file 2 [file Supplementary_file_2.docx]

**Supplementary Figure S1.** Univariable logistic regression analysis of factors associated with neurological improvement after spinal cord injury. Patients with an increase of at least one AIS grade at 1 year were classified as the improvement group, whereas those with no AIS grade change or death were classified as the non-improvement group. Odds ratios (ORs) and 95% confidence intervals (CIs) are presented for each variable.

| Variable | OR (95% CI) | *P* value |
| --- | --- | --- |
| ISS | 0.824 (0.778–0.873) | <0.001 |
| Lumbar spinal cord injury | 3.593 (1.631–7.913) | 0.002 |
| Lumbar vertebral fracture | 3.185 (1.383–7.337) | 0.006 |
| Cervical spinal cord injury | 0.417 (0.220–0.791) | 0.007 |
| Male sex | 0.382 (0.184–0.791) | 0.01 |
| Cervical vertebral fracture | 0.504 (0.278–0.913) | 0.024 |
| ANC (×10⁹/L) | 0.863 (0.801–0.929) | <0.001 |
| WBC (×10⁹/L) | 0.872 (0.813–0.934) | <0.001 |
| MONO (×10⁹/L) | 0.239 (0.105–0.543) | <0.001 |
| MLR | 0.354 (0.193–0.649) | <0.001 |
| SIRI | 0.929 (0.895–0.964) | <0.001 |
| Age (years) | 0.959 (0.936–0.982) | <0.001 |
| AISI | 1.000 (0.999–1.000) | <0.001 |
| NLR | 0.964 (0.934–0.994) | 0.018 |
| SII | 1.000 (1.000–1.000) | 0.011 |
| AIS grade A | 0.032 (0.013–0.079) | <0.001 |

**Supplementary Figure S2.** Variable selection using least absolute shrinkage and selection operator (LASSO) regression based on neurological improvement at 1 year. Patients with an increase of at least one AIS grade were defined as the improvement group, whereas those with no AIS grade change or death were classified as the non-improvement group. **(A)** LASSO coefficient profiles of candidate variables. **(B)** Selection of the optimal penalty parameter (λ) based on 10-fold cross-validation. The dotted vertical lines indicate the values of λ corresponding to the minimum mean cross-validated error and the one-standard-error criterion.
